# The Knowledge Sharing Capability in Innovative Behavior: A SEM Approach from Graduate Students’ Insights

**DOI:** 10.3390/ijerph20021284

**Published:** 2023-01-10

**Authors:** Víctor Yepes, Salvador López

**Affiliations:** Institute of Concrete Science and Technology (ICITECH), Universitat Politècnica de Valencia, 46022 Valencia, Spain

**Keywords:** knowledge sharing capability, knowledge sharing facilitators, innovative behavior, innovation climate, graduate students, SEM

## Abstract

The capability to share knowledge is considered one of the most relevant components of knowledge management. Moreover, there is little empirical evidence indicating how future human resources in the construction industry value the richness of knowledge sharing and the richness of their innovative behavior. The purposes of this study are (1) to determine which facilitators, from the point of view of master’s degree students related to engineering and construction management in Spain, most substantially influence knowledge sharing capability; (2) to test whether knowledge sharing capability (KS) positively influences innovative behavior (IB); and (3) demonstrating whether organizational innovation climate (OIC) is a factor that moderates the relationship between KS and IB. In this research, we have proposed a theoretical model and empirically tested the model in a sample of 253 master’s degree students in public universities in Spain. The findings support the proposed model, and the structural equation modeling (SEM) evaluation suggests that, among all the facilitators of KS, information and communication technologies (ICT) stand out among the other facilitators and have a more significant influence on KS. Furthermore, the research found a direct correlation between KS and IB and causal links between OIC and IB.

## 1. Introduction

Beyond the advances in science and technological development, the main driving force of companies is their human resources and how managers manage knowledge. The problem is not the generation of knowledge in the construction sector but the waste of valuable information for future projects [1]. It is a reality that future construction professionals must go beyond traditional technical training to project themselves into a challenging future [2]. The frameworks of innovation systems and changes in the organizational culture of Spanish construction firms corroborate the above. See, for example, “Creative innovation in Spanish construction firms” [3]. Therefore, evaluating their future vision and knowing their strengths and weaknesses is essential. This research addresses three fundamental issues: the facilitators of knowledge sharing capabilitie (KS) and their link to innovative behavior (IB) under the moderation of an organizational climate conducive to innovation.

Previous studies, summarized below, have demonstrated the importance of KS facilitators. For example, Lin [4] found that the convergence between individual, organizational and technological factors positively influenced KS. Kumar and Rose [5] identified seven facilitators of KS, which they grouped into two categories. The first group is called individual factors (pleasure in sharing knowledge to help others, reciprocity, knowledge self-efficacy, self-image). The second group is called organizational factors (the norms favorable to sharing, generalized trust, and reward systems). Sáenz et al. [6] extended the study of individual and organizational factors. They approached the former from the perspective of personal interaction. To the latter, they added management processes and delved into ICT-based technological factors. The results suggest that many facilitators take advantage of ICTs, but this does not imply that their use is fundamental to KS.

Knowledge gaps indicate that only some components of the model have been analyzed by the literature separately. However, the literature has not analyzed organizational innovation climate (OIC) as a moderating variable between KS and IB. In addition, most studies need to address the views of graduate students.

**Figure 1:**
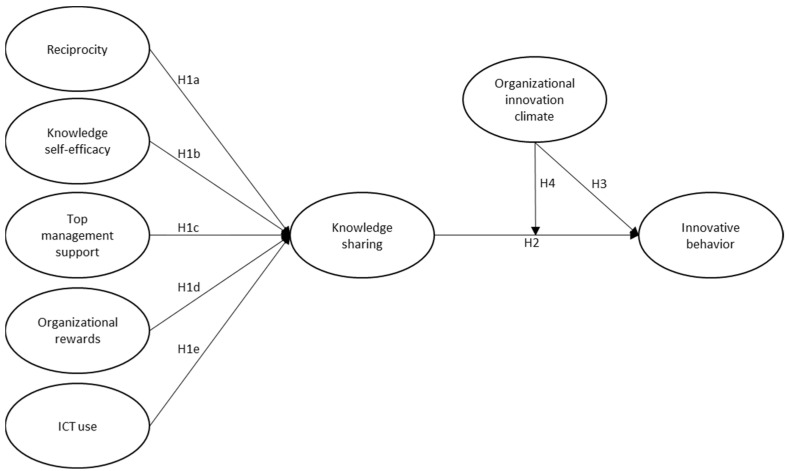
Conceptual framework. H1a: Reciprocity is positively related to KS; H1b: Knowledge self-efficacy is positively related to KS; H1c: Top management support is positively associated with KS; H1d: There is a significant relationship between rewards and KS; H1e: Information and communication technologies have a positive impact on KS. H2: Employee knowledge sharing positively influences their innovative behavior; H3: Innovative climate is positively associated with employees’ innovative behavior; H4: The OIC positively moderates the relationship between knowledge sharing and employees’ innovative behavior.

For further empirical evidence and a more profound understanding, this paper will examine the moderation role of the OIC in the KS-IB relationship and include reciprocity in KS facilitators. We conducted this study in the context of an educational setting linked to the construction industry with the objectives of (1) determining which facilitators, from the point of view of master’s degree students related to construction engineering and management in Spain, most substantially influence the ability to exchange knowledge; (2) testing whether KS positively influences innovative behavior; and (3) demonstrating whether an OIC is a factor moderating the KS-IB relationship. Figure 1 shows the model proposed in the research.

Thus, to fill the above research gaps, the present study was undertaken to elucidate some of the following research questions:

RQ1. Which facilitator of KS is most important for graduate students?

RQ2. How does KS influence IB?

RQ3. What role does OIC play between KS and IB?

In order to answer the previous research questions, this research implements structural equation modeling (PLS-SEM) to examine the correlation between the components of the research model from data collected from a survey administered to 253 students from public universities in Spain (See Table A1). We chose the survey as a research tool because of its suitability for collecting the beliefs of a large number of individuals [7]. The students’ insights on every question supplied information for further analysis. We expect our study to provide theoretical perspectives on the vision of potential workers in the sector, as well as practical implications for improving the IB of human resources in organizations.

## 2. Research Model and Hypotheses Development

### 2.1. Reciprocity

From the organizational context, the literature review indicates a need to explore further the reciprocity derived from KS [4,8,9,10,11]. Although this area has received little attention, several authors have pointed out the relevance of reciprocity for successful KS [12,13,14]. That is, regardless of the professional or academic setting, reciprocal relationships are more fruitful than unidirectional ones [15]. In other words, reciprocity in KS creates a feeling of participation capable of transforming staff attitudes into IB [15].

From the educational context, Su and Zhang [16] found that it is essential for graduate students to actively seek multiple feedback channels to stimulate innovative behavior. Endres and Chowdhury [8] examined that expected reciprocity in KS depended on individual skill level, positive team attitudes, and demographic diversity. Encouraging reciprocity may have positive effects, but poor team attitudes, perceived low ability, and lesser people diversity counteract them.

### 2.2. Knowledge Self-Efficacy and Knowledge Sharing

Kankanhalli et al. [9] defined knowledge self-efficacy as the confidence of staff to contribute valuable and correct knowledge to an organization. It is a self-assessment of their ability to successfully organize and perform daily tasks [8]. When employees are more confident in their ability to contribute knowledge, they are more prone to share valuable knowledge [4,9,11,17,18]. Wipawayangkool and Teng [19] discovered that workers with a stronger sense of self-efficacy tended to share their knowledge both voluntarily and at the request of others. In contrast, Masa’deh’s [20] study found that knowledge self-efficacy did not significantly influence employees’ ability to share knowledge.

At the educational level, Su and Zhang [16] found that students’ self-efficacy highly depends on the personality and diversity of instructor dynamics, which is essential for enhancing knowledge self-efficacy. From these personality traits of academics, students can develop knowledge-based self-efficacy.

### 2.3. TOP Management Support and Knowledge Sharing

Recent studies have examined the impact of top management support on KS. For example, Lee et al. [21] further explored the link between top management support and KS. The results showed that top management is a critical element in building knowledge-sharing solid groups, and its importance can be identified by developing organizational policies that drive appropriate resources. Lo et al. [22] addressed the relationships between individual factors, organizational factors, KS, and business performance. Their results indicated that organizational factors associated with top management support and incentives boosted knowledge donation and knowledge-gathering processes. Although most research corroborated the significant impact of top management support on KS, several studies have revealed contradictory results [23]. These results imply that more than top management support is needed to contribute directly to KS.

According to Eletter et al. [24], from an academic context, the role of the teacher is fundamental to engaging students in KS, and the way to achieve this is through group interaction. Combining these activities with an online environment has been recognized as efficient for KS among students [24,25]. For this research, the role of a teacher is comparable to that of a manager in an organization.

### 2.4. Organizational Rewards and Knowledge Sharing

Organizational culture and rewards are positively associated with knowledge sharing [26]. Many studies have focused on the role of rewards and KS. First, motivation theory highlights the role of rewards in employees’ behavior toward KS [26]. Other studies have empirically examined the effects of rewards in organizations [18,27,28,29]. Moon and Park [28] found that organizational rewards motivate people to contribute knowledge, but the quality of knowledge is also in question. Kankanhalli et al. [9] found a positive relationship between organizational reward and knowledge contribution. Eletter et al. [24] suggested that balancing individual and group rewards can facilitate KS. Therefore, when employees consider the benefits of KS important, their willingness to share knowledge increases [4,9,11,17,18,30].

On the other hand, some studies claim that reward systems have a negative effect. Bock and Kim [30] stated that expected rewards discourage positive attitudes toward KS in the organization’s context. In another study, Bock et al. [17] found that extrinsic rewards sometimes negatively influence attitudes toward KS. Lin [4] found an insignificant relationship between extrinsic rewards and KS intention. Finally, employees are more likely to engage in KS activities when they perceive a link between KS behavior and rewards, such as promotions, salary increases, and career advancement [24].

### 2.5. ICT Usage and Knowledge Sharing

Different experts recognize the importance between KS and ICT in their research. For example, Islam and Ashif [31] indicated that ICT could facilitate collaborative work and knowledge transfer. Ibrahim et al. [32], Mazzucchelli et al. [33], Roberts [34] and Safdar et al. [35] discovered that ICT enhances KS by reducing temporal and spatial barriers between people and improving access to knowledge. ICTs streamline the collection, storage, and sharing of knowledge on a scale that was impossible until recently, which strengthens the process and ability to share knowledge [31,33,34,36,37].

In the context of education, ICT has revolutionized the methods of teaching and learning. According to Jain and Gupta [27], the new generation is rapidly adjusting to technology and is enthusiastic about learning through technological tools, which benefits knowledge and its transfer. These results converge with other studies [23,27]. Other studies have also shown that ICT [38,39], mainly social networks, is essential in facilitating KS [25,40,41]. ICT fosters interaction and communication among students. Wangpipatwong [42] found that technical support towards students and knowledge-sharing among fellow peers positively influence their knowledge-sharing behavior, leading to IB.

This study suggests the following hypotheses in light of a thorough evaluation of the literature on knowledge sharing enablers:

**H1a.**  *Reciprocity is positively related to KS.*

**H1b.**  *Knowledge self-efficacy is positively related to KS.*

**H1c.**  *Top management support is positively associated with KS.*

**H1d.**  *There is a significant relationship between rewards and KS*.

**H1e.**  *Information and communication technologies have a positive impact on KS.*

### 2.6. Knowledge Sharing and Innovative Behavior

Recently, there has been increasing interest in studying the relationship between KS and IB [43,44]. Several studies agree that KS predicts employees’ innovative behavior [43,45,46,47,48,49]. Mura et al. [45] argued that staff perceptions could influence this relationship. Radaelli et al. [46] added that employees who shared knowledge were more likely to change their innovative behavior. Udin et al. [43] added that sharing generates trust and communication among workers [47], which triggers innovative behaviors.

KS enhances employees’ ability to innovate because information must go through an internalization process [44,50] to make it available to recipients [51]. That is, the process helps employees acquire and build a more valuable knowledge, which undoubtedly modifies both innovative thinking and behavior. Zhang et al. [44] found that KS among employees generates more ideas and strategies, ultimately stimulating innovative behavior.

Based on the above review, we propose the following hypothesis:

**H2.**  *Employee knowledge sharing positively influences their innovative behavior.*

### 2.7. Organizational Innovation Climate and Innovative Behavior

The OIC is a precursor [52] and predictor [53] of IB in individuals. On the one hand, it is an indicator of organizational functioning, teamwork, and collective learning [44,54]. On the other hand, it reflects whether managerial encouragement of innovation influences staff attitudes [17,54,55]. A successful OIC combines autonomy, flexibility, trust, cooperation, and communication [55,56].

Several studies have examined the correlation between OIC and IB [52,53,57,58]. Most of them agree with the arguments of Jaiswal and Dhar [59], Khalili [56], Ren and Zhang [60] that OIC is essential for staff behavior to be more innovative [61]. You et al. [62] recently added to their research the mechanisms underlying employees’ innovative behavior at organizational, individual, and work levels. Their results confirm the positive role of OIC on the innovative behavior of individuals.

From an educational perspective, Wang et al. [63] demonstrated that elements including an open academic environment, social support and guidance, a team of tutors, and innovation outcomes significantly impacted graduate students’ innovative behavior. On the other hand, educational studies are scarce compared to the industrial sector [64]. However, students’ behavior is not significantly different from workers’ behavior because both are strongly related to human and environmental factors [35]. Therefore, these references provide good support for assuming that an innovative climate is positively associated with employees’ innovative behavior; hence, we hypothesize the following:

**H3.**  *Innovative climate is positively associated with employees’ innovative behavior.*

### 2.8. Moderating Role of Organizational Innovation Climate

Individuals may feel less psychologically confident in the face of new challenges or technologies and are more likely to embrace conservative strategies instead of innovative behaviors [65]. An organization’s innovation climate is critical to reversing this situation. A good environment creates psychological safety for employees to accept new ideas, share their knowledge, and take on new challenges [44]. As a result, this secure feeling reduces the fear of failure and its adverse effects [66] and increases an individual’s willingness to innovate. Yu et al. [57] found that KS and OIC had a positive relationship with IB.

On the other hand, the study by Witherspoon et al. [36] took the opposite view, in which they emphasized that human factors have their vulnerable side, thus weakening the OIC in its moderating role between KS and IB. This failure corresponds to information hoarding, competitive use of knowledge for personal gain [67], and self-interest [17], which overcome the innovation climate.

Therefore, we hypothesize the following:

**H4.**  *The OIC positively moderates the relationship between knowledge sharing and employees’ innovative behavior*.

## 3. Materials and Methods

### 3.1. Participants

The dataset consisted of 253 usable responses from graduate students in master’s degree programs related to engineering and construction management in Spain. It is a convenience sample, non-probabilistic, due to not having full access to a nationwide random sample [7]. Therefore, this sample includes only those participants the authors could access directly, plus some snowball effect. According to the responses to the questionnaire, we were able to profile them as follows: under 27 years of age (54.9%), male (56.9%), and with a maximum of three years of professional experience (66.8%) and having a previous working background with a contractor (52.2%). As for their academic background, 68.4% of them were civil engineers, 19.8% were architects, and the rest identified themselves as building engineers. These are the usual classifications of graduate students in construction programs [68].

### 3.2. Questionnaire Survey

The questionnaire consisted of four distinct parts: (1) respondent characterization, (2) KS enablers, (3) innovation climate factors, and (4) elements that shape innovative behav-ior. The first part included professional title, gender, and age questions. The items consid-ered for the second part focused on the five second-order latent variables (reciprocity, knowledge-self efficacy, top management support, organizational rewards, and ICT use). The third and fourth parts of the questionnaire corresponded to the OIC (3 items) and IB (3 items), respectively. We developed the 24 items considered in the questionnaire from a thorough literature review based on [14,20,29,32,44,57].

### 3.3. Measurement

Reflective measurement models have been the instrument of countless studies. However, applying these traditional models under some circumstances is often less fruitful [69]. Our study addressed the construction of KS using a formative-formative hierarchical component model (HCM). Model construction was achieved under the conformation of five lower-order latent variables shaped by 18 formative indicators, ultimately generating a higher-order component [70]. We operationalized the KS as a second-order formative construct comprising five first-order formative constructs: reciprocity, knowledge self-efficacy, top management support, organizational rewards, and ICT use (as shown in Table 1). The items were scored on a seven-point Likert scale ranging from 1 (strongly disagree) to 7 (strongly agree). We adopted two three-item measures from [57] to assess OIC and the remaining ones from Ibrahim et al. [32] to measure IB, as shown in Table 2. All outcome variables were measured reflectively using the same seven-item scale.

We took the following steps to lessen the impact of variance bias/common method bias (CMV). First, we separated the endogenous variables from the exogenous variables. Second, we randomized the items, and finally, we performed a pretest. Like Hulland et al. [71], we took these steps to minimize order effects and response bias.

## 4. Data Analysis

Statistical Package for the Social Sciences (SPSS) version 25 [72] and Smart PLS 3 was used for our statistical analyses [73]. We selected component-based PLS-SEM above co-variance-based structural equation modeling (CB-SEM) for the following reasons: (a) the structural model incorporates one or more formative constructs (constructs are operation-alized as composites); (b) the structural model is complex with several indicators, con-structs, and path relationships; (c) the research goal is to identify critical constructs [74,75]; and (d) the moderating effect of OIC needs to be assessed, so PLS-SEM is adequate, as it provides path analysis estimates using reduced error terms [76].

This study’s data analysis was divided into two sections. The measuring approach was first tested to ensure that the formative and reflective constructs were reliable and valid. Second, the structural model trajectories (significance and path coefficients) were evaluated with 5000 resampling iterations using the bootstrapping approach [76].

### 4.1. Convergent and Discriminant Validity

Convergent validity represents the degree of agreement between different indicators of the same construct. When factor loadings, CR, and AVE coefficients surpass 0.5, this measure is feasible [76]. Table 2 confirms the unidimensionality and authenticity of the composites’ convergent validity, as their indices exceed 0.50.

### 4.2. Testing Measurement Model

The external weights and *p*-values for the first-order (formative) dimensions are the primary recommendations for evaluating formatively modeled HOCs [76,77]. Table 1 represents the KS (second-order formative construct), its dimensions (first-order formative), and the assessment of their external weights and *p*-values. In addition to the above, the same table should contain the variance inflation factor (VIF) to check for multicollinearity issues. According to Becker et al. [77] and Hair et al. [76], VIF values lower than five do not imply multicollinearity problems. In our study, there are no such problems. On the other hand, OIC showed the highest contribution to IB (β = 0.431; *p* < 0.01), followed by KS (β = 0.262; *p* < 0.01).

### 4.3. Significance of Structural Model

The statistical significance of path coefficients between exogenous and endogenous variables is investigated using structural models [76]. A PLS-SEM algorithm and a boot-strapping procedure assess the significance level of structural relationships using path coefficients and t-values by drilling down into the model [76]. Path coefficients provide the standardized β coefficients and t-values. The former are derived from regression, while the latter determine the significance level of the study constructs if the value of 1.64 is exceeded [76,78].

## 5. Results

The first factors to be evaluated in formative models are collinearity and the relevance of the formative indicators. The VIF is used to assess collinearity, as described in the previous section. Our study’s PLS algorithm results revealed no multicollinearity issues because all scores were less than three [75]. We could determine the relevance of the formative indicators after investigating the significance of the external weights and loadings in our model. Furthermore, the bootstrapping results revealed that ICT use was the only statistically significant weight (*p* < 0.01) among the proposed formative indicators. It implies that its items made an adequate contribution to the formative construct. Finally, Table 1 displays the size and significance of the weights calculated using the bootstrapping procedure with 5000 subsamples.

The loading of each reflective indicator on its corresponding construct (>0.7), composite reliability value (CR > 0.7), Cronbach’s alpha (>0.7), average variance extracted value (AVE > 0.5), and Fornell–Larcker criteria were used to evaluate the reflective measurement model [76,79]. The reflective measurement model demonstrated that the indicators’ properties exceeded the theoretical ranges. Furthermore, the square roots of the AVE values were more significant than the internal construct correlations, and the heterotrait-monotrait ratio (HTMT) values were less than 0.85 [80] (see Table 2).

The results reveal the predictive relevance of IB through the direct relationships between KS and OIC without the interaction effect. Using the PLS-SEM blindfolding procedure, the predictive relevance of the model was confirmed. The corroboration parameter is the Stone-Geisser value, where a Q^2^ > 0 represents that it meets the parameter [74]. For our study, the value of Q^2^ = 0.300 represents its validity. To confirm the suitability of the PLS-SEM model for the standardized value, we employed the root-mean criterion (SRMR < 0.08) [78], which for our study, is below the theoretical threshold (SRMR = 0.060) (see Table 3). Furthermore, the results reveal a coefficient of determination (R^2^) of 41.9, which means that KS and OIC collectively explain 41.9% of the variance of IB (see Table 4).

### 5.1. Testing of Hypotheses

Table 4 summarizes the coefficients of the structural path model produced by the PLS-SEM bootstrapping technique. The model shows the direct effects of KS and OIC. The relationship shows that KS has a highly significant and positive impact on innovative behavior (β = 0.262; *t* = 3.217; *p* < 0.01). Therefore, H2 is accepted. In addition, the innovation climate in the organization (β = 0.431; *t* = 5.808; *p* < 0.01) also shows a significant and positive effect on innovative behavior.

### 5.2. Moderation Effects

**Figure 2:**
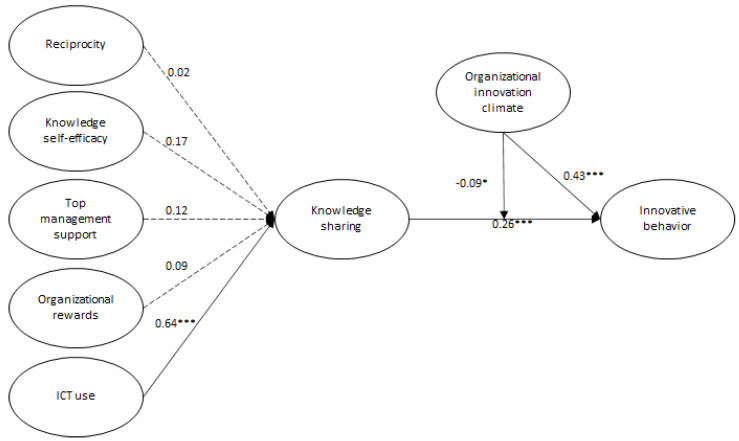
Results of the proposed model. Notes: Dashed lines indicate insignificant paths: *** *p* < 0.001; * *p* < 0.05.

We examined in the paper the effect of OIC on the relationship between KS (second-order formative construct) and IB (see Figure 1 and Figure 2). OIC (β = −0.085; *t* = 2.116; *p* < 0.01) establishes a significantly negative moderation on the relationship between KS and IB. When interaction effects are introduced into the model, the coefficient of determination changes (R^2^). Table 4 shows that the OIC interaction increases the R^2^ value from 0.419 to 0.429. This increase means that the variance explanation for IB is improved by introducing the interaction effect of OIC. According to Hair et al. [76], the R^2^ represents, as insignificant as it may seem for moderation, an essential role in the interaction effect.

The model’s effect size (f^2^) measures the accuracy with which the exogenous variables predict the endogenous variables. According to Aguinis et al. [81], the effect size ranges between 0.02, 0.15, and 0.35. Where f^2^ = 0.02 denotes a minor effect, f^2^ = 0.15 is a medium effect, and f^2^ = 0.35 is a significant effect. Table 4 shows that OIC has the most substantial effect on IB (f^2^ = 0.150), followed by the KS effect on IB (f^2^ = 0.055), which has the slightest effect. Similarly, the effect size of the interaction term, i.e., OIC (f^2^ =0.018) is limited. Aguinis et al. [81] suggested that moderators with even lower effects cannot be ignored. Therefore, the study’s H4 is also accepted.

## 6. Discussion

This study joins recent efforts that call attention to the theoretical and methodological distinctions between formative and reflective measurement models to address the lack of formative measurement models in KS research [69].

One of the goals of this study was to determine, from the viewpoint of graduate students enrolled in master’s degree programs in engineering and construction management in Spain, which facilitators had the most significant influence on the capabilities to exchange knowledge. A second objective was empirically determining whether KS positively impacted innovation behavior from the respondents’ perspective. Finally, it was necessary to demonstrate whether the relationship between KS and innovation behavior was supported by environments that promote innovation.

Structural equation model analysis of the first set of hypotheses confirmed that, among the group of knowledge facilitators, only ICT use had a statistically significant effect on KS. Table 4 presents results indicating that respondents believe KS is most affected by ICT use. The remaining predictors of KS were not statistically significant for the respondents. This research complements previous research by hypothesizing that the future construction workforce relies on the use of ICT to consolidate their ability to share knowledge, which is very different from what the literature shows at the organizational level, as noted below.

The results revealed that reciprocity had no significant effect on KS. Therefore, they are not consistent with the arguments of Caimo and Lomi [12], Richards [13], and Tamjidyamcholo et al. [14]. We suggest that to ensure a sense of reciprocity, it is necessary to engage in KS; the leader figure should constantly work on team attitudes and focus on transforming individual goals into shared goals and increasing capabilities [8].

Knowledge self-efficacy was not found to be a factor that significantly impacts KS. This result is not consistent with the arguments of Bock et al. [17], Casimir et al. [18], Kankanhalli et al. [9], Lin [4], Nguyen et al. [11], Wipawayangkool and Teng [19], Kumar and Rose [5], and Lavanya [82]. Our results support the approach of Masa’deh et al. [20], who argued that knowledge self-efficacy does not significantly influence people’s ability to exchange knowledge. From an organizational point of view, top management should provide helpful information to boost employees’ knowledge self-efficacy. For example, hiring proactive employees with high cognitive ability, self-esteem, and developed interpersonal skills can generate a workforce with high self-efficacy [20]. From an educational point of view, the focus should be on teachers’ personalities and cognitive-dynamic strategies to improve cognitive self-efficacy.

Our research finds that top management support does not significantly impact KS. Therefore, it is not consistent with the arguments of Lo et al. [22], Meddour et al. [83], Lin [4], Kumar and Rose [5], Masa’deh et al. [20], and Mueller [84], from the organizational aspect. It is also different from the academic context according to the arguments of Eid and Al-Jabri [25] and Eletter et al. [24], who concluded that teachers were essential to consolidate group interactions among students and favor KS. Our results are consistent with the findings of Lo and Tian [23], who argue that many studies yield contradictory results and that intermittent top management support does not directly reinforce KS. Regardless of the academic or organizational context, we suggest that skills, continuous and systematic work and leadership support are crucial in influencing people’s KS.

The results revealed that rewards did not correlate significantly with KS. They support the arguments of Bock and Kim [30], Bock et al. [17] and Lin [4], who argued that expected rewards inhibit positive attitudes toward KS. Therefore, based on our study, we infer that individuals begin to react to expected rewards and are less willing to exchange knowledge if their demands are not met. When someone receives rewards continuously, the incentive function becomes an obligation.

The results showed a positive and significant correlation between ICT use and KS. These findings are consistent with the claims made by Lin [4], Saenz et al. [6], Ibrahim et al. [32], Mazzucchelli et al. [33], Roberts [34], Safdar et al. [35], Islam and Ashif [31], Ruikar et al. [37] and Ryan et al. [85] that ICT can help with the codification, integration, and transfer of organizational knowledge. From a scholarly perspective, it is consistent with the claims made by Eid and Al-Jabri [25], Eid and Nuhu [38], Kaba and Ramaiah [39], Moghavvemi et al. [40] and Sharabati [41] that social media are the primary enablers of KS.

The results showed that KS is a predictor of IB. They support the arguments of Akhavan et al. [48], Hansen [47], Kim et al. [49], Mura et al. [45], Radaelli et al. [46] and Udin et al. [43]. As a result, managers in both the construction and education sectors should actively reinforce individuals’ perceptions of KS in order for them to share knowledge. Individual KS will increase participation, which will increase knowledge internalization. On the other hand, OIC also shows a significant and positive effect on IB. These findings support the claims made by Jaiswal and Dhar [59], Khalili [56], Ren and Zhang [60], Shanker et al. [58] and You et al. [62] that OIC is a central aspect of employees’ innovative behavior. Education research is scarce compared to the industrial sector [64]. However, students’ conduct is similar to that of organizational members, as individual behavior is often related to human and environmental factors [35].

Our results also showed that OIC did not have a positive or significant moderating effect. This conclusion reinforces the arguments of Witherspoon et al. [36], Chow et al. [67] and Bock et al. [17]. They argued that the ineffectiveness of OIC is a product of the dark side of human factors such as knowledge hoarding, competitive use, and self-interest.

## 7. Conclusions

The results of the measurement model test, including convergent validity, discriminant validity, variance inflation factor, and explanatory power, were satisfactory. The new generations recognize that among all the enablers studied, the use of ICT is the primary way to strengthen KS in the future of the construction sector. However, from the authors’ perspective, one of the main problems is idealization of ICT as an “end product” and not as a tool to expand into new horizons. The innovative behavior of individuals must be based on more than the exclusive use of ICTs, since ICTs alone are not synonymous with innovation. It is necessary to combine principles such as reciprocity, management support, and knowledge self-efficacy with new technologies so that they become the elements that transform both institutions and individuals.

Moreover, the importance of the educational sector is emphasized since it is a fact that it is rapidly adapting to the use of new technologies in teaching. The educational sector should maintain sight of fundamental aspects such as the development of reciprocity, self-efficacy of knowledge, and developing social and creative skills in its educational programs. On the other hand, it must continue on the path of adaptation to new technologies. With these two aspects strengthened, education can transform the innovation culture of professionals to the extent of permeating it in the sector’s organizations when they enter the workplace.

From the respondents’ perspective, KS and OIC are precursors to IB. It is clear that the behavior of individuals depends on human and environmental factors, regardless of their professional background. At the organizational level, future human resources in the sector must be immersed in an environment that allows for development, tolerates failure, and provides the means to open up the possibilities for IB. Organizations will likely transform openness to innovation if they work systematically on OIC.

Finally, our study corroborates that OIC is not a factor that enhances the bidirectional relationship between KS and IB. We authors agree that deeper issues of individuals’ behavior need to be delved into to reduce self-interested knowledge hoarding.

### Limitations and Future Research

Although the work adds knowledge and value to the literature, it also has certain limitations. First, the cross-sectional design does not eliminate the possibility of a long-term causal correlation emerging due to changes in the psychology and confidence of individuals over time. Second, we derived facilitators from studies conducted in a professional setting before applying them in an educational setting. In addition, most respondents had less than three years of professional experience, which would condition the study’s results. It should be added that the topics addressed from the professional environment in students were with the firm intention of analyzing the students’ perception of these topics. A longitudinal study would overcome this limitation. Future research could replicate this study in other countries and combine quantitative and qualitative data, not only among students but also among teachers, to strengthen the view of the impact of KS on IB and add other facilitators of KS. In addition, future studies could examine how other variables may act as moderators or mediators of KS, e.g., organizational culture or the facilitating conditions between the dependent and independent constructs. Finally, the literature requires more research linking students’ vision to the work environment and understanding, from the beginning, what to do with human resources to improve the innovation aspect in the construction sector.

## Figures and Tables

**Table 1:** Formative constructs and their statistical characteristics.

HOC	LOC	Weights	VIF
Knowledge sharing (KS)	Knowledge self-efficacy (KSE)	0.168 **	2.458
	Confidence in one’s capacity (KSE 1)	0.641 ***	1.436
	Confidence in one’s own experience (KSE 2)	0.222 *	1.415
	Importance of sharing one’s knowledge (KSE 3)	0.177 *	1.192
	Confidence in the capacity of others (KSE 4)	0.270 **	1.237
	Top management support (TMS)	0.116	1.770
	Promote knowledge sharing (TMS 1)	0.361 ***	1.347
	Support for knowledge sharing (TMS 2)	0.038 ns	1.475
	Resources for knowledge sharing (TMS 3)	0.599 ***	1.524
	Interest in welfare after knowledge sharing (TMS 4)	0.259 *	1.436
	Organizational rewards (OR)	0.064	2.049
	Knowledge Sharing is rewarded with a higher salary (OR 1)	0.362 ***	1.278
	Knowledge Sharing is rewarded with bonuses (OR 2)	0.339 ***	1.144
	Knowledge Sharing is rewarded with promotion (OR 3)	0.607 ***	1.275
	Reciprocity (REC)	0.119	2.856
	Share ideas only if others reciprocate (REC 1)	0.199 *	1.551
	Share knowledge only if there is a response (REC 2)	0.205 *	1.623
	Sharing knowledge benefits everyone (REC 3)	0.399 **	1.754
	Transform attitude into innovative behavior (REC 4)	0.433 **	1.681
	Information and communications technology use (ICT)	0.635 ***	2.968
	Use of electronic storage (such as online databases and data warehouses) (ICT 1)	0.414 ***	1.719
	Use knowledge networks (such as groupware, intranet, and virtual communities) (ICT 2)	0.382 ***	1.566
	Internal use of technology in the organization (ICT 3)	0.418 ***	1.465

Notes: *** *p* < 0.01; ** *p* < 0.05; * *p* < 0.1; ns = Not significant; VIF = variance inflation factor; HOC = Higher order constructs; LOC = Lower order constructs/items.

**Table 2:** Statistical values of the reflective constructs.

Constructs	Loadings	α	CR	AVE
Organizational innovation climate (OIC)		0.805	0.885	0.719
Free environment to work creatively (OIC 1)	0.828 ***			
Dedication of budget to develop innovative projects (OIC 2)	0.724 ***			
Tolerance to failures (OIC 3)	0.730 ***			
Innovative behavior (IB)		0.844	0.905	0.760
Search for new ways to put ideas into practice (IB 1)	0.894 ***			
Search for new solutions to solve problems (IB 2)	0.860 ***			
Search for new methods, techniques, or work tools (IB 3)	0.637 ***			

Notes: Each of the loadings was significant (*** *p* < 0.01); α = Cronbach’s alpha; AVE = average variance extracted; CR = construct reliability coefficient.

**Table 3:** Manifest variables and their PLS prediction.

Items	PLS RMSE	Q^2^_predict_	LM RMSE	PLS-LM RMSE
IB 2	1.511	0.253	1.527	−0.016
IB 3	1.548	0.124	1.551	−0.003
IB 1	1.269	0.309	1.279	−0.010
OIC 1	1.107	0.466	1.114	−0.007
OIC 2	1.495	0.320	1.509	−0.014
OIC 3	1.545	0.342	1.549	−0.004

Notes: RMSE = root mean squared error; PLS = partial least squares path model; LM = linear regression model.

**Table 4:** Findings from the PLS-SEM analysis.

Hypothesized Relationships	β	*t*- Statistics	*p*- Values	95% Lower	CI Upper	Effect Size (f^2^)	R^2^ Value	Q^2^ Value	SRMR	Hypothesis Supported
H1a: Reciprocity → Knowledge sharing capability	0.12	0.635	0.263	−0.186	0.428					No
H1b: Knowledge self-efficacy → Knowledge sharing capability	0.17	1.154	0.124	−0.072	0.409					No
H1c: Top management support → Knowledge sharing capability	0.12	0.965	0.167	−0.078	0.315					No
H1d: Organizational rewards → Knowledge sharing capability	0.09	0.707	0.240	−0.129	0.308					No
H1e: ICT use → Knowledge sharing capability	0.635	3.703	0.000	0.322	0.887					Yes
H2: Knowledge sharing capability → Innovative behavior	0.262	3.217	0.001	0.141	0.394	0.055	0.419	0.300	0.06	Yes
H3: Organizational innovation climate → Innovative behavior	0.431	5.808	0.000	0.295	0.534	0.150				Yes
H4: Moderating effect: Organizational innovation climate → Innovative behavior	−0.085	2.116	0.034	−0.152	−0.020	0.018	0.429			Yes

## Data Availability

Not applicable.

## Appendix A

**Table A1:** Measurement Scales Developed by the CFA.

Construct	Factor	Dimension	Description	Source
Knowledge Self-Efficacy	KSE	KSE 1	Confidence in one’s capacity.	Adapted from: [20]
		KSE 2	Confidence in one’s own experience.	
		KSE 3	Importance of sharing one’s knowledge.	
		KSE 4	Confidence in the capacity of others.	
Top Management Support	TMS	TMS 1	Promote knowledge sharing.	Adapted from: [20,22]
		TMS 2	Support for knowledge sharing.	
		TMS 3	Resources for knowledge sharing.	
		TMS 4	Interest in welfare after knowledge sharing.	
Organizational Rewards	OR	OR 1	Knowledge Sharing is rewarded with a higher salary.	Adapted from: [20,30]
		OR 2	Knowledge Sharing is rewarded with bonuses.	
		OR 3	Knowledge Sharing is rewarded with promotion.	
Reciprocity	REC	REC 1	Share ideas only if others reciprocate.	Adapted from: [15,29]
		REC 2	Share knowledge only if there is a response.	
		REC 3	Sharing knowledge benefits everyone.	
		REC 4	Transform attitude into innovative behavior.	
ICT Use	ICT	ICT 1	Use of electronic storage (such as online databases and data warehouses).	Adapted from: [20]
		ICT 2	Use knowledge networks (such as groupware, intranet, and virtual communities).	
		ICT 3	Internal use of technology in the organization.	
Organizational Innovation Climate	OIC	OIC 1	Free environment to work creatively.	Adapted from: [57]
		OIC 2	Dedication of budget to develop innovative projects.	
		OIC 3	Tolerance to failures.	
Innovative Behavior	IB	IB 1	Search for new ways to put ideas into practice.	Adapted from: [32]
		IB 2	Search for new solutions to solve problems.	
		IB 3	Search for new methods, techniques, or work tools.	

Notes: The scale used is a seven-point Likert scale ranging from 1 (strongly disagree) to 7 (strongly agree).
